# Injectable Thermosensitive Hydrogel Containing Bakuchiol Reduces Periodontal Inflammation and Alveolar Bone Loss in a Rat Model

**DOI:** 10.3390/jfb16080292

**Published:** 2025-08-13

**Authors:** Seong-Jin Shin, Gyu-Yeon Shim, Seong-Hee Moon, Yu-Jin Kim, Hyun-Jin Kim, Seunghan Oh, Jung-Hwan Lee, Ji-Myung Bae

**Affiliations:** 1 Department of Dental Biomaterials, College of Dentistry, Wonkwang University, 460 Iksan-daero, Iksan City 54538, Republic of Korea; ko2742@naver.com (S.-J.S.); koala9710@naver.com (G.-Y.S.); shmoon@jif.re.kr (S.-H.M.); shoh@wku.ac.kr (S.O.); 2 Institute of Tissue Regeneration Engineering (ITREN), Dankook University, Cheonan City 31116, Republic of Korea; yujin911031@daum.net (Y.-J.K.); ducious@gmail.com (J.-H.L.); 3 Department of Oral Anatomy and Dental Research Institute, College of Dentistry, Wonkwang University, 460 Iksan-daero, Iksan City 54538, Republic of Korea; khjin1005@wku.ac.kr; 4 Institute of Biomaterials Implant, Wonkwang University, 460 Iksan-daero, Iksan City 54538, Republic of Korea; 5 Musculoskeletal and Immune Disease Research Institute, Wonkwang University, 460 Iksan-daero, Iksan City 54538, Republic of Korea

**Keywords:** bakuchiol, thermosensitive hydrogel, ligature-induced periodontitis, rat model, alveolar bone loss, periodontal disease, anti-inflammatory medicaments

## Abstract

This study aimed to develop and evaluate a bakuchiol-loaded thermosensitive hydrogel (BTH) as a novel local drug delivery system for the management of periodontitis. Bakuchiol, a natural phenolic compound extracted from *Psoralea corylifolia*, was incorporated into a hydrogel composed of poloxamers and carboxymethylcellulose. The gelation behavior, physicochemical properties, and drug release profile were analyzed. Additionally, antibacterial activity against *Porphyromonas gingivalis* was assessed. Cytotoxicity was evaluated in human gingival fibroblasts and RAW 264.7 cells. Anti-inflammatory effects were determined by measuring proinflammatory cytokine expression in lipopolysaccharide-stimulated RAW 264.7 macrophages. Furthermore, alveolar bone loss, cytokine expression, and histological findings were assessed in a rat model of ligature-induced periodontitis. BTH demonstrated sol–gel transition at body temperature, with sustained drug release over 15 days. Moreover, it exhibited significant antibacterial activity against *P. gingivalis* and was non-cytotoxic at an extract concentration of 6.25%. In vitro, it significantly downregulated inflammatory cytokines in activated macrophages. In vivo, BTH application reduced alveolar bone loss and interleukin-1β expression in gingival tissues. Histological analysis confirmed decreased inflammatory cell infiltration and alveolar bone destruction. Thus, BTH demonstrated both antibacterial and anti-inflammatory activities, exhibiting potential as a promising therapeutic strategy for localized periodontal treatment.

## 1. Introduction

Periodontitis, a major inflammatory disease of the oral cavity, affects approximately 20–50% of the global population, making it a significant public health concern [1,2]. This condition causes progressive destruction of the tooth-supporting periodontal tissues, potentially leading to tooth mobility and eventual loss [3]. In addition to its local effects, periodontitis has emerged as a key contributor to systemic inflammation and is strongly associated with various chronic disorders, including cardiovascular diseases, diabetes, neurodegenerative conditions, and cancer [4,5,6,7].

Mechanical debridement remains the primary management approach for removing bacterial plaque from tooth surfaces; however, accessing deep periodontal pockets and root furcations can be challenging [8]. Systemic medications such as antibiotics, nonsteroidal anti-inflammatory drugs (NSAIDs) are often used as adjuncts to mechanical therapy or in cases of deep pockets and aggressive periodontitis [9]. Although commonly prescribed, systemic antibiotics and NSAIDs have notable drawbacks, including disruption of the microbiome, the emergence of antibiotic-resistant bacteria, and gastrointestinal side effects [10].

Local drug delivery systems have emerged as a promising alternative to address the limitations of systemic treatment, particularly in cases of deep periodontal pockets or cases where the local drug concentration needs to be increased with minimal systemic side effects [11]. Among these, injectable thermosensitive hydrogels have shown promise because of their ability to transition from a sol at room temperature to a gel at a physiological temperature, enabling precise and sustained drug delivery into deep periodontal pockets [12]. These hydrogels can incorporate antibiotics, anti-inflammatory agents, and/or natural compounds and have resulted in improvements in clinical parameters such as probing depth and attachment levels [11].

Many natural products and naturally occurring phenolic compounds have shown antibacterial and anti-inflammatory effects in the management of periodontitis [13,14,15]. Among them, bakuchiol, a meroterpene phenol derived from *Psoralea corylifolia*, has recently gained attention for its therapeutic potential [16]. Traditional Chinese and Indian medicine regimens have long utilized bakuchiol for its antitumor and anti-inflammatory properties [17]. In the fields of dermatology and esthetics, it has emerged as a well-tolerated alternative to retinol, demonstrating promising antibacterial, anti-inflammatory, and antiaging properties [18]. In the field of dentistry, it has been reported to show antimicrobial effects against dental caries-causing bacteria such as *Streptococcus mutans* [19,20]. Furthermore, it has reportedly shown antimicrobial effects against *Porphyromonas gingivalis*, a causative agent for periodontal disease [21]. However, further research is required to develop dental materials that utilize the antimicrobial and anti-inflammatory properties of bakuchiol.

**Figure 1:**
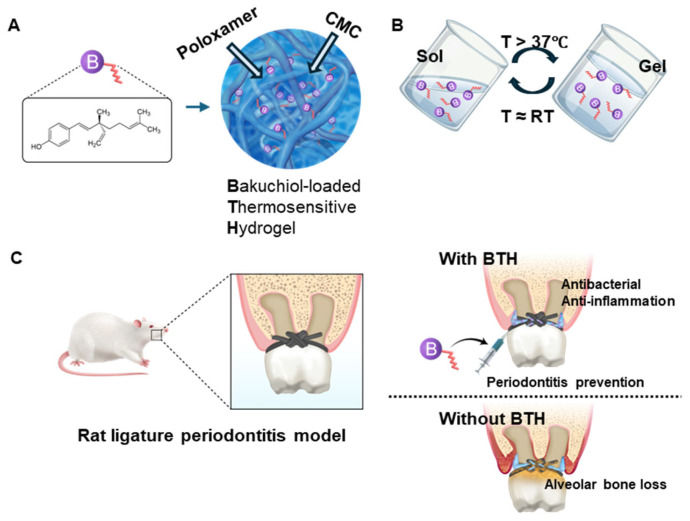
Experimental design and materials. (**A**) Preparation of a bakuchiol-loaded thermosensitive hydrogel (BTH). (**B**) Thermosensitive properties of BTH. (**C**) Flowchart showing prevention of periodontitis by BTH in a rat model.

The aim of this study was to develop and evaluate a bakuchiol-loaded thermosensitive hydrogel (BTH) for local drug delivery to reduce periodontal inflammation. Our formulation utilized two types of poloxamers to precisely control the gelation temperature. We investigated the key biological parameters essential for periodontitis management, particularly focusing on the antibacterial and anti-inflammatory activities as well as the physical properties of BTH (Figure 1). To our knowledge, this is the first investigation of a bakuchiol-based hydrogel for the management of periodontitis via dual antibacterial and anti-inflammatory effects. The null hypothesis was that antibacterial and anti-inflammatory effects, as well as the reduction of alveolar bone loss, would not show any significant differences between a BTH application group and a control group.

## 2. Materials and Methods

### 2.1. Preparation of BTH

To prepare a hydrogel with a gelation temperature suitable for use in the oral cavity, various gels (EX 1, EX 2, EX 3, and EX 4 BTH) with different contents were prepared by adjusting the amount of poloxamer. Their compositions are summarized in Table S1.

Because bakuchiol is not water-soluble, a solution was prepared by mixing 2 mL of bakuchiol (purity > 98%; Habotech Co., Ltd., Nanjing, China), 10 mL of polyethylene glycol (average Mn = 400; Sigma-Aldrich, St. Louis, MO, USA), and 10 mL of ethanol (EMSURE^®^, Merck, Darmstadt, Germany). A 2 mL aliquot of this bakuchiol solution was then added to 18 mL of phosphate-buffered saline (PBS; pH 7.4; Gibco, Carlsbad, CA, USA) to yield a final concentration of 0.9 vol% in the hydrogel. To impart thermosensitivity, 15 wt% Pluronic^®^ F-127 (PF-127; Sigma-Aldrich) was added, and the gelation temperature was achieved by adding 0–7.5 wt% poloxamer 188 (P188; Sigma-Aldrich, St. Louis, MO, USA). The desired viscosity was achieved by adding 1 wt% carboxymethylcellulose sodium salt (CMC; medium viscosity; Sigma-Aldrich, St. Louis, MO, USA). For preparation of BTH, the components were mixed in a 100 mL glass bottle on a magnetic stirrer (r100; Yoke, Shanghai, China) for 24 h in a 4 °C refrigerator. To test the intrinsic effects of bakuchiol, a hydrogel without bakuchiol was prepared as a vehicle control.

### 2.2. Rheological Characteristics, Gelation Temperature, pH, and Scanning Electron Microscopy (SEM) Features of the Hydrogel

Rheological measurements were performed using a Discovery HR-1 (TA Instruments, Newcastle, USA) equipped with a 25.0-mm parallel Peltier plate. The elastic modulus (G′) and viscous modulus (G″) were analyzed with a 1 mm gap at a fixed frequency of 1.0 Hz. The sol–gel transition temperature for the samples was measured within a range from 4 °C to 45 °C at a rate of 1 °C/min. For measurement of the gelation temperature, 2 mL of the hydrogel in a glass container was placed in a temperature-controlled heating bath (RW3-0525, Jeiotech, Daejeon, Republic of Korea) at 4 °C for 20 min. The gelation temperature at which the solution stopped flowing was recorded by tilting the glass container at a 45° angle and increasing the temperature by 1 °C every 5 min [22]. The pH of the hydrogel was measured using a pH meter (Orion 3-Star; Thermo Scientific, Chelmsford, MA, USA). On the basis of the optimal rheological behavior of EX3 BTH for oral application, this formulation was selected for subsequent experiments.

To confirm whether the components were well mixed, we analyzed the samples using Fourier transform infrared spectroscopy (FTIR; Vertex-70V/Hyperion 3000, Bruker Optic GmbH, Ettlingen, Germany). To further understand the structure of the hydrogel by surface evaluations, we freeze-dried BTH using a freeze dryer (LYB-8604; Operon Advantech, Gimpo, Republic of Korea). Following platinum vacuum coating using an ion sputter coater (108 Auto; Cressington Scientific Instruments Ltd., Watford, UK), the surface was observed using SEM (JSM-6360; JEOL, Tokyo, Japan) at 500× magnification.

### 2.3. Bakuchiol Release

A 500 uL aliquot of BTH stored at 4 °C was placed in a dialysis kit (PURD60050, Sigma-Aldrich, St. Louis, MO, USA). The kit containing the gel was then placed in a 50 mL plastic specimen bottle containing 40 mL of PBS (Gibco). The bottle was stored in a 37 °C shaking incubator (JSSI-100C, JSR, Gongju, Republic of Korea) at 25 rpm. The eluted solution in PBS was replaced with fresh PBS daily for 15 days. The absorbance of the removed solution was measured in a 96-well UV microplate (Corning Inc., Corning, NY, USA) using a spectrophotometer (Spectramax Mini; Molecular Devices, San Jose, CA, USA) to detect bakuchiol release at 263 nm, which is the peak absorbance of bakuchiol. The values were converted into a percentage of the total bakuchiol concentration [23].

### 2.4. Antibacterial Activity

*P. gingivalis* (ATCC 53978) was obtained from the American Type Culture Collection (ATCC; Manassas, VA, USA). The bacteria were cultured in 3% brain heart infusion (BHI; BD Biosciences, Bergen, NJ, USA) supplemented with 50 μg/mL of hemin (Sigma-Aldrich) and 0.5 μg/mL of menadione (Sigma-Aldrich) in an anaerobic incubator (Whiley DG250 Workstation, Don Whiley Scientific, Bingley, UK) under 37 °C, 10% H_2_, 10% CO_2_, and 80% N_2_.

An agar diffusion test was performed to evaluate the antibacterial activity of BTH against *P. gingivalis*. The bacterial suspension was diluted to an optical density of 0.1 at 600 nm, and 100 μL of the bacterial suspension (1 × 10^7^ colony-forming units) was inoculated on blood agar. The blood agar was prepared using 3% BHI with 5% defibrinated sheep blood (Synergy Innovation Co., Ltd., Seongnam, Republic of Korea) and 1% agar (BD Biosciences). Sterilized paper discs (ADVANTEC, Toyo Roshi Kaisha Ltd., Tokyo, Japan) with a 6-mm diameter were placed on the agar plates. Then, 20 μL each of BTH and the vehicle gel were applied to individual paper discs. The positive control was 0.12% chlorhexidine (Hexamedine; Bukwang Pharm. Co., Seoul, Republic of Korea), and the negative control was PBS (Gibco); 5 μL of each was applied to each paper disc. After 48 h of culture in the anaerobic incubator, the major and minor axes of the inhibition zone were measured using vernier calipers (CD-15CP; Mitutoyo, Kawasaki, Japan), and the mean value was obtained. The antibacterial activity was determined as the mean value excluding the 6-mm disc diameter.

### 2.5. Cell Viability and Anti-Inflammatory Effect of BTH

All cells used in the experiment were cultured using Dulbecco’s Modified Eagle Medium (Gibco) supplemented with 10% fetal bovine serum (Gibco) and 100 units/mL of penicillin–streptomycin (Hyclone, Logan, UT, USA) in a 5% CO_2_ incubator at 37 °C. BTH or vehicle gel (0.5 g) was placed in a 15 mL tube. After allowing gelation at 37 °C for 30 min, culture medium (5 mL) was added for hydrogel extraction at a ratio of 0.1 g/1 mL. The extraction was continued for 24 h at 37 °C. Human gingival fibroblast (HGF-1, CRL-2014™) and RAW 264.7 cell lines were used and obtained from ATCC. HGF-1 cells and RAW 264.7 cells were seeded at 5 × 10^3^ and 2 × 10^5^ cells/well, respectively, in 96-well plates. After 24 h of seeding, diluted gel extracts were added to the cells. Cell viability was measured after 24 h using a CCK-8 kit (Dojindo, Kumamoto, Japan).

To determine whether the BTH extract possessed anti-inflammatory properties, RAW 264.7 cells were seeded at 5 × 10^5^ cells in 24-well plates. After 24 h, diluted BTH extract (at noncytotoxic concentrations) and lipopolysaccharide (LPS; from *Escherichia coli*; Sigma-Aldrich) at a concentration of 1 ng/mL were added to the cells. After 24 h, RNA was extracted using a Ribospin kit (Geneall, Seoul, Republic of Korea). Real-time polymerase chain reaction (PCR) was performed using the Exicycler 96 Real-Time Quantitative Thermal Block (Bioneer, Daejeon, Republic of Korea) to quantify the expression of inflammation-related genes (tumor necrosis factor alpha [TNF-α], interleukin-1 beta [IL-1β], interleukin 6 [IL-6], and interleukin 10 [IL-10]). The used primer sequences are listed in Table S2.

### 2.6. Alveolar Bone Loss and Inflammatory Cytokine Expression in a Rat Model of Ligature-Induced Periodontitis

The protocol for the animal experiment was approved by the Institutional Animal Care and Use Committee of Wonkwang University (WKU 21-51). Twenty-four 7-week-old male Sprague-Dawley rats (Samtako BIO KOREA, Osan, Republic of Korea) weighing 180–220 g were used for the animal experiment. The rats were randomly divided into four groups (*n* = 6/group; three animals/cage) and housed at room temperature (23 °C ± 2 °C) with a relative humidity of 50% ± 20% and a 12 h/12 h controlled dark/light cycle. The rats were provided with food and water ad libitum.

**Figure 2:**
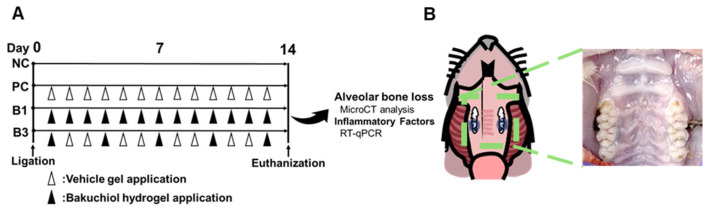
Schematic diagram of the timeline and the rat model of ligature-induced periodontitis. (**A**) Experimental timeline: NC, negative control group without ligation; PC, positive control group with tooth ligation and daily application of vehicle gel; B1, experimental group with tooth ligation and daily BTH application; B3, experimental group with tooth ligation and BTH application every 3 days and vehicle gel application on the intervening days. (**B**) Illustration and photograph showing ligature of the maxillary second molars on both sides in a rat.

The four groups were as follows (Figure 2A):

NC: Negative control group without ligation

PC: Positive control group with tooth ligation and daily application of the vehicle gel

B1: Experimental group with tooth ligation and daily BTH application

B3: Experimental group with tooth ligation and BTH application every 3 days and vehicle gel application on the intervening days

After 7 days of adaptation, the rats, except those in the negative control group, were anesthetized with an intraperitoneal injection of chloral hydrate (Sigma-Aldrich, 400 mg/kg) for tooth ligature. To induce periodontitis, a 4-0 silk ligature (AILEE Co., Ltd., Busan, Republic of Korea) was applied around the maxillary second molars on both sides, and a surgeon’s knot was tied on the buccal side [24] (Figure 2B). Starting 1 day after ligature, 25 μL of the gel was applied to each ligatured side (50 μL in total) daily using an oral Zonde needle (JD-S-124-70-20G, Jeung Do Bio & Plant Co., Ltd., Seoul, Republic of Korea). BTH was applied daily in the B1 group and every 3 days in the B3 group, with vehicle gel applied on the intervening days in the latter group. In the positive control group, the vehicle gel was applied daily. Before gel application, the rats were anesthetized with isoflurane (HANA PHARM Co., Hwaseong, Republic of Korea).

Fourteen days after ligature, the rats were sacrificed by CO_2_ inhalation, and their maxillae were isolated and fixed with 4% paraformaldehyde (Geneall, Seoul, Republic of Korea) at 4 °C, which was replaced with PBS (Gibco) before micro-computed tomography (micro-CT). A micro-CT device (SKYSCAN 1076; Bruker, Kontich, Belgium) for laboratory animals was used at a tube voltage of 100 kV and a tube current of 100-μAm, with a resolution of 18 μm. Cross-sectional micro-CT images were reconstructed in three dimensions using CTvox (Bruker, Kontich, Belgium). Micro-CT measurements were conducted in a blinded manner. The distance between the cementoenamel junction and alveolar bone crest on the mesial and distal sides of the ligatured second molar was measured to evaluate bone loss using ImageJ software (v1.8, NIH, Bethesda, MD, USA).

For analysis of inflammatory cytokines, gingival tissue around the second molar was biopsied immediately after sacrifice and placed in TRIzol solution (Invitrogen, Carlsbad, CA, USA). The tissue was then homogenized using a BioMasher II homogenizer (Nippi, Tokyo, Japan) for several minutes. cDNA was synthesized using the Omniscript Reverse Transcriptase Kit (Qiagen, Germantown, MD, USA), and 10 pmol of each primer (Table S2) was mixed with Greenstar qRT-PCR Master Mix (Bioneer). Real-time PCR analysis was performed using the Exicycler 96 Real-Time Quantitative Thermal Block (Bioneer) to quantify IL-1β gene expression; GAPDH mRNA was used for normalization. The used primer sequences are listed in Table S2.

### 2.7. Histological Analysis

After micro-CT analysis, the specimens were decalcified for 2 weeks using a 1:1 mixture of 8% hydrochloric acid (Avantor, Radnor Township, PA, USA) solution and 8% formic acid (Daejung Chemicals & Metals Co., Ltd., Siheung-Si, Republic of Korea) solution. The decalcification solution was replaced after 1 week. Paraffin blocks were prepared and cut into 4.0-μm-thick slices for slide preparation. After hematoxylin and eosin (H&E) staining, the slides were observed under a microscope at 40× and 100× magnifications to identify the histopathological characteristics. Epithelial cell migration, chronic inflammatory cells, capillary vasodilation, and alveolar bone resorption were observed.

### 2.8. Statistical Analysis

The sample size for the animal experiment was determined using a sample size calculator [25]. Statistical analyses were performed using SPSS software (SPSS 26; Chicago, IL, USA). One-way analysis of variance was performed for the analysis of bakuchiol release, the antibacterial activity of the hydrogel, cytotoxicity, alveolar bone loss, and inflammatory factor expression, with post hoc analysis using Tukey’s honestly significant difference test. The significance level was set at *p* < 0.05.

## 3. Results

### 3.1. Rheological Characteristics, Gelation Temperature, pH, and SEM Analysis of the Hydrogel

**Figure 3:**
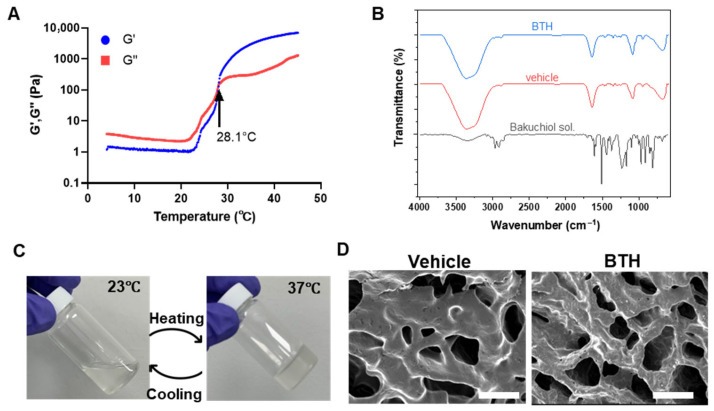
Physicochemical characteristics of the bakuchiol-loaded thermosensitive hydrogel (BTH). (**A**) Rheological analysis of BTH, including evaluation of the elastic modulus (G′) and viscous modulus (G″). (**B**) Fourier transform infrared spectroscopy (FTIR) analysis of BTH: vehicle, hydrogel without bakuchiol; Bakuchiol sol, bakuchiol mixed with ethanol and PEG. (**C**) Thermosensitive properties of BTH. BTH exists as a solution at room temperature but transforms into a solid gel at body temperature. (**D**) Scanning electron microscopy images of freeze-dried BTH. Surface images of BTH and the vehicle show a mesh-like network in the hydrogel. Scale bar = 50 μm.

For the induction of thermosensitive properties, whereby the hydrogel changed from a solution at room temperature to a gel at body temperature, BTH was fabricated using poloxamers as a base. The rheological properties varied with temperature according to the poloxamer ratio. As the P188 content increased relative to the PF127 content, the lower critical solution temperature (LCST) increased (Figure S1). EX3 BTH showed an LCST of 28.1 °C, as verified by the rheometer (Figure 3A), and exhibited a neutral pH range (Table S3).

The optimal rheological behavior of EX3 BTH made it the material of choice for subsequent experiments. FTIR analysis confirmed that bakuchiol was well mixed in the formulation, as evidenced by the peak shift in BTH (Figure 3B). When stored at 37 °C, proper gelation was observed (Figure 3C). After freeze-drying, SEM examination of the surface structure revealed the porous structure characteristic of hydrogels (Figure 3D).

### 3.2. Bakuchiol Release from BTH

**Figure 4:**
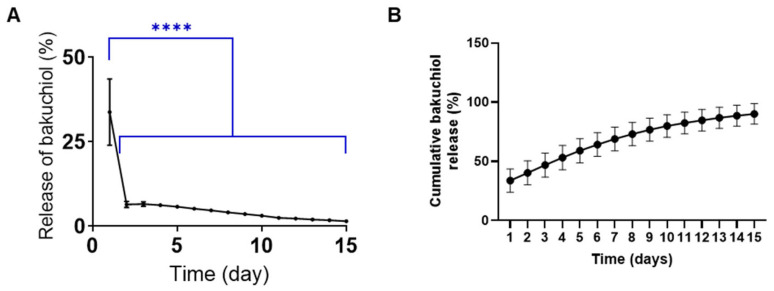
Release profile of the bakuchiol-loaded thermosensitive hydrogel (BTH). (**A**) Time-dependent release of BTH. Release of bakuchiol from BTH over time is the highest on the first day, followed by consistent release thereafter. Statistical analysis was performed using post hoc Tukey’s test (****: *p* < 0.001). (**B**) Cumulative bakuchiol release from BTH. The graph demonstrates the continuous release of bakuchiol over a 15-day period, demonstrating the sustained-release profile of the hydrogel formulation.

Analysis of the release profile revealed that BTH exhibited an initial burst release of 33.8% bakuchiol on the first day, followed by consistent sustained release over 15 days (Figure 4A,B). This biphasic release pattern was considered advantageous for periodontal management because it would provide immediate therapeutic action through the initial burst release and maintain an effective concentration through sustained release. This controlled-release system would enhance the bioavailability of bakuchiol at the disease site by ensuring its prolonged presence in the periodontal pocket, addressing both acute inflammation and long-term disease management.

### 3.3. Antibacterial Activity of BTH and Its Effects on Cell Viability

**Figure 5:**
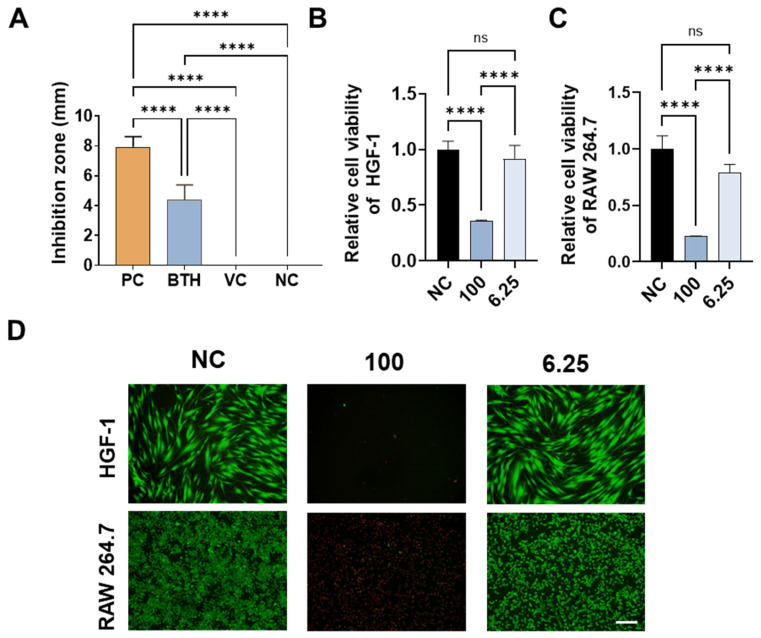
Biological properties of the bakuchiol-loaded thermosensitive hydrogel (BTH). (**A**) Antibacterial effects against *Porphyromonas gingivalis*. The inhibition zones demonstrate the antibacterial activity of the hydrogel against *P. gingivalis*. PC, positive control (chlorhexidine); VC, vehicle gel as vehicle control; NC, negative control (phosphate-buffered saline). (**B**) Cell viability evaluation using the CCK assay shows the effects of BTH on human gingival fibroblast (HGF-1) cells. NC, negative control (cells only). (**C**) Cell viability evaluation using the CCK assay shows the effects of BTH on RAW 264.7 cells. NC, negative control (cells only). (**D**) Live and dead staining images. Scale bar = 200 μm. Statistical analysis was performed using analysis of variance with Tukey’s honestly significant difference test as a post hoc test (****: *p* < 0.0001, ns: not significant). NC, negative control (cells only); 100, BTH extract; 6.25, 1/16 dilution of BTH extract.

To evaluate the antibacterial activity of BTH against *P. gingivalis*, a major bacterial species responsible for periodontal disease, the agar diffusion method was employed to measure the antimicrobial activity of the hydrogel. BTH showed clear antibacterial activity against *P. gingivalis*, although the inhibition zone was smaller than that observed with chlorhexidine, which was the positive control (*p* < 0.0001) (Figure 5A). No antibacterial activity was observed for the negative control, PBS, or vehicle gel without bakuchiol.

To investigate the effect of BTH on cells relevant to the periodontal environment, experiments were conducted using gingival fibroblasts and macrophages, both of which play critical roles in periodontal health and disease. BTH was extracted at a concentration of 0.1 g/mL for these cellular experiments. The undiluted extract showed cytotoxicity when applied directly to the cells. However, when diluted to 1/16 of the original concentration (6.25%), no toxicity was observed (Figure 5B–D).

### 3.4. Anti-Inflammatory Properties of BTH

**Figure 6:**
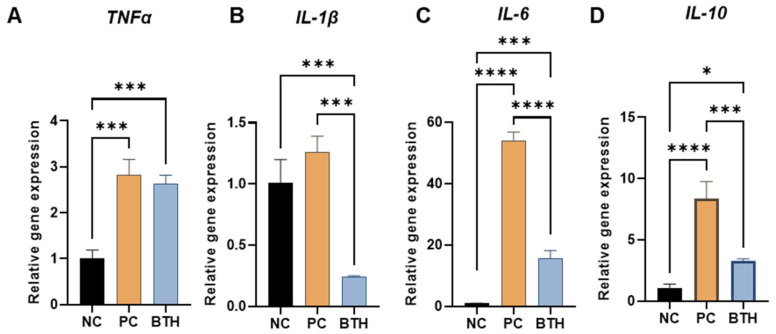
Anti-inflammatory properties of the bakuchiol-loaded thermosensitive hydrogel (BTH). The graphs show that BTH reduced lipopolysaccharide-induced inflammation in RAW 264.7 cells. (**A**) TNF-α, tumor necrosis factor alpha; (**B**) IL-1β, interleukin-1 beta; (**C**) IL-6, interleukin 6; (**D**) IL-10, interleukin 10. Statistical analysis was performed using analysis of variance with Tukey’s honestly significant difference test as a post hoc test (*: *p* < 0.05, ***: *p* < 0.001, and ****: *p* < 0.0001). NC, negative control (cells only); PC, LPS 1 ng/mL; BTH, LPS with BTH extract.

In the inflammatory response experiments, murine macrophages (RAW 264.7 cells) were stimulated with LPS to induce inflammation. When these LPS-stimulated cells were treated with diluted BTH extract, significant reductions were observed in the expression of inflammatory cytokines, including IL-1β (*p* < 0.001) (Figure 6B), IL-10 (*p* < 0.001) (Figure 6C), and IL-6 (*p* < 0.0001) (Figure 6D).

### 3.5. Alveolar Bone Loss, Inflammatory Cytokines, and Histology in a Rat Model of Ligature-Induced Periodontitis

**Figure 7:**
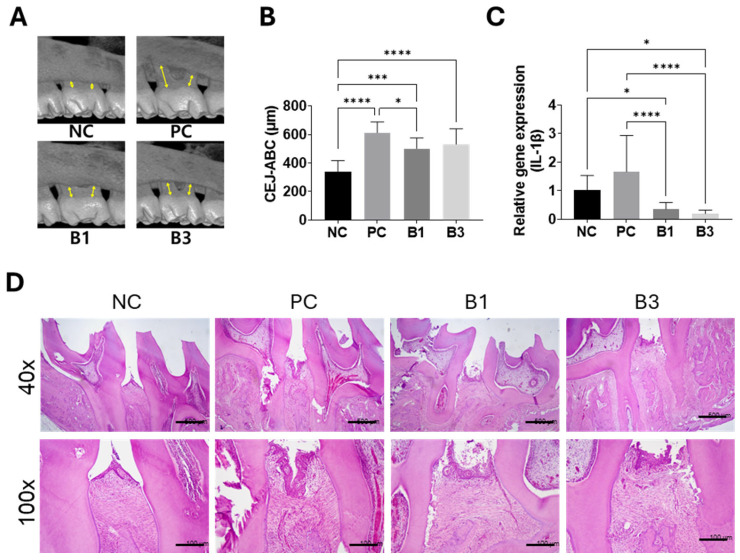
In vivo study using the bakuchiol-loaded thermosensitive hydrogel (BTH) in a rat model of ligature-induced periodontitis. (**A**) Micro-computed tomography (micro-CT) images. The yellow arrows indicate the measured distance between the cementoenamel junction (CEJ) and the alveolar bone crest (ABC), which represents alveolar bone loss. (**B**) Alveolar bone loss measurements. The distance between CEJ and ABC was measured using micro-CT. (**C**) Inflammatory cytokine expression. The graph shows the expression levels of the proinflammatory cytokine interleukin-1 beta (IL-1β) in rat gingival tissue. (**D**) Histological analysis. Representative hematoxylin and eosin-stained images show histological changes in periodontal tissues across different groups. Data were statistically analyzed using one-way analysis of variance with Tukey’s honestly significant difference test as a post hoc test for multiple comparisons (*: *p* < 0.05, ***: *p* < 0.001, and ****: *p* < 0.0001).

To determine the preventive effect of BTH on alveolar bone loss, the distance between the cementoenamel junction and alveolar bone crest was measured on micro-CT images (Figure 7A). Compared with the NC group, the ligature groups showed significant differences in bone loss (*p* < 0.001) (Figure 7B). The B1 group showed significantly less bone loss than did the PC group (*p* < 0.05), whereas the B3 and PC groups showed no significant difference from the PC group (*p* > 0.05).

The IL-1β expression level in the gingival tissue around the ligatured area in the PC group was significantly higher than that in the NC group (*p* < 0.05), whereas the IL-1β expression levels in the B1 and B3 groups were not significantly different from that in the NC group (*p* > 0.05) (Figure 7C).

To analyze the effect of BTH on the tissue around the ligatures, H&E staining was performed (Figure 7D). Compared with the epithelial cells in the NC group, those in the PC group proliferated and migrated toward the root, with infiltration of chronic inflammatory cells such as lymphocytes and plasma cells, capillary vasodilation, and alveolar bone resorption. Although the B1 and B3 groups also showed epithelial cell proliferation, chronic inflammatory cell infiltration, and alveolar bone resorption, these findings were not as severe as those in the PC group.

## 4. Discussion

In this study, a novel BTH was developed to reduce periodontal inflammation. BTH demonstrated significantly higher antibacterial and anti-inflammatory activities, as well as a superior ability to reduce alveolar bone loss, compared with the negative control. Therefore, the null hypothesis was rejected. The EX3 BTH was selected because it showed suitable rheological characteristics and a gelation temperature between room temperature and body temperature, as confirmed by rheometry. Moreover, it exhibited antibacterial activity against *P. gingivalis* and demonstrated no cytotoxicity at concentrations that showed anti-inflammatory effects. Finally, daily application of BTH for 14 days in a rat model of ligature-induced periodontitis showed inhibitory effects on inflammation and alveolar bone loss.

The antibacterial activity of bakuchiol is attributed to its ability to inhibit DNA synthesis by degrading bacterial DNA helices and inhibiting the topoisomerase II enzyme [26]. This mechanism underlies its efficacy against several oral bacteria, such as *S. mutans*, *Streptococcus sobrinus*, *P. gingivalis, Enterococcus faecalis*, and *Lactobacilli* [19]. Son et al. reported sustained antibacterial activity against *S. mutans* by mixing bakuchiol with experimental fluoride varnish [20]. To the best of our knowledge, no prior study has investigated the antibacterial activity of a hydrogel mixed with bakuchiol. In the present study, BTH demonstrated significant antibacterial activity against *P. gingivalis*, the primary pathogen in chronic periodontitis, whereas a vehicle gel without bakuchiol exhibited no antibacterial effects.

In the ligature-induced periodontitis rat model, daily application of BTH (B1 group) significantly reduced bone loss compared with the PC group. However, bone loss in the B3 group, where BTH was applied every 3 days, was not significantly different from that in the PC group, despite BTH releasing a high concentration of bakuchiol for up to 3 days. This outcome may be attributed to a limitation of the ligature-induced periodontitis model, in which BTH was applied to teeth with silk ligatures. In clinical practice, medication is administered after scaling and root planing to prevent inflammation. However, the silk ligatures in this model may have caused persistent inflammation, potentially reducing the effects of BTH. Despite this limitation, the inhibition of bone loss by BTH in the B1 group is a finding of significant clinical importance.

We used a model of ligature-induced periodontitis in this study because it can simulate bone loss caused by the deposition of dental calculus. Braided silk sutures were used to facilitate more significant food deposition. The ligature was applied to maxillary teeth because alveolar bone loss occurs more rapidly in the maxilla than in the mandible [24]. The maxillary second molars were selected to enhance ligature retention, as these teeth have more interproximal contacts than the first and third molars. Given that inflammation and bone loss do not occur around ligated rat teeth in sterile conditions, the accumulation of oral bacteria at the ligature site is considered the primary cause of periodontitis in this model [27]. In silk ligature-induced periodontitis, alveolar bone loss occurs without the application of orthodontic force, unlike models using elastic bands or coil springs [28].

The anti-inflammatory effects of bakuchiol and its underlying mechanisms have been previously studied. Kumar et al. demonstrated, using enzyme-linked immunosorbent assay, that bakuchiol reduces LPS-induced inflammatory cytokines (IL-6, TNF-α, IL-1β, interferon gamma) in RAW 264.7 cells. They further established that this effect is associated with the nuclear factor kappa–light-chain-enhancer of activated B cells (NF-κB) pathway [29]. Similarly, BTH application reduced the expression of IL-1β, IL-6, and IL-10 in LPS-stimulated RAW-264.7 cells. Additionally, Zhao et al. revealed that bakuchiol can modulate LPS-induced lung injury by targeting TLR4/MyD88/NF-κB and Keap1/Nrf2/HO-1 pathways [30]. Because periodontitis is also induced by gram-negative bacterial LPS, it may be potentially regulated through similar mechanisms; this is supported by the in vivo findings of this study, where BTH application resulted in decreased IL-1β levels. The expression levels of IL-1β in the B1 and B3 groups were lower than those in the PC group. As a critical proinflammatory cytokine, IL-1β plays an essential role in the immune and inflammatory response and osteoclastogenesis [31]. Previous studies have shown elevated levels of IL-1β, TNF-α, and matrix metalloproteinase-9 in gingival tissues from ligature-induced periodontitis models [32,33]. Because IL-1β is an inflammatory factor involved in acute inflammation, additional factors such as IL-8, which is involved in chronic inflammation, and receptor activator of nuclear factor kappa-Β ligand, which is associated with bone loss, need to be evaluated in future studies.

The poloxamers PF127 and P188 were used to induce thermosensitivity in the hydrogel. Poloxamers are highly temperature-sensitive polymers with a polyethylene oxide–b-polypropylene oxide–b-polyethylene oxide structure [34]. The gel is formed as hydrophilic polyethylene oxide blocks create a micelle structure at specific concentrations and temperatures [35]. Poloxamers are highly stable and do not cause toxicity or irritability, even in acid or base solutions [36]. PF127 has been used in drug delivery agents because of its anticancer, anti-inflammatory, antibacterial, and wound-healing effects [37]. Because PF127 is prone to instability in the aqueous phase with low mechanical strength, an additional polymer should be added [38].

In this study, BTH was prepared for application in gingival pockets. The mean temperature in the gingival pocket is (36.6 °C ± 0.4 °C) [39]. Because the desired gelation temperature, similar to that in the gingival pocket, could not be achieved using PF127 alone, P188 was added. The gelation temperature increased with higher concentrations of P188. The optimized composition of BTH comprising 15 wt% PF127 and 5 wt% P188 allowed for easy syringeability at room temperature and gelation within the gingival pocket. In contrast, the vehicle gel without bakuchiol did not undergo gelation upon temperature increase. CMC was incorporated to enhance adhesiveness. The carboxyl groups in CMC are likely to form hydrogen bonds with mucin glycoproteins on oral mucosal surfaces, promoting adhesion to gingival tissues and prolonging the hydrogel’s residence time, despite the challenges posed by salivary flow [40].

In addition to bakuchiol, various compounds derived from natural sources have been investigated for the treatment of periodontitis. Resveratrol, a polyphenolic compound with anti-inflammatory and antioxidant effects, has shown therapeutic efficacy in animal models of periodontitis, although it was not delivered via a controlled-release system [41]. The incorporation of other phytochemicals, such as curcumin and berberine, into hydrogels for the management of periodontitis has been previously reported [23,42]. These natural products exhibit antibacterial or anti-inflammatory activity; however, their inherent yellow color implies potential staining or discoloration of the teeth or gingiva when applied in the oral cavity [43,44]. In contrast, bakuchiol is colorless at the applied concentration and poses no risk of staining. Moreover, as bakuchiol is widely used as a cosmetic ingredient, its effects on the human body are well known, with few reported side effects [16,18].

In this study, bakuchiol was released for up to 15 days from BTH. Although bakuchiol is a natural compound, its duration of sustained release was comparable to that of a polyvinyl alcohol–chitosan hydrogel loaded with metronidazole, which showed a released duration of 14 days [45], or longer than that of a doxycycline-loaded gel, which showed a release duration of 7 days [46]. Previous animal studies on thermosensitive gels for periodontitis showed improvement when the gel was administered every 3 days [12,47]. However, differences in ligature material, duration, and injection site across studies may account for variations in treatment outcomes, even when drug release profiles are similar. From a translational standpoint, BTH has strong potential as an injectable local therapy because of its ease of administration, thermosensitive gelation near body temperature, and sustained release profile.

This study had some limitations. First, although BTH was released over 15 days in vitro, daily application was necessary to prevent bone loss in the animal experiment. This suggests that the hydrogel does not remain at the application site for an adequate duration. Second, we used an animal model of acute ligature-induced periodontitis, which may not reflect the chronic, multifactorial nature of human periodontitis. Third, the specific molecular pathways for the antibacterial and anti-inflammatory effects of bakuchiol remain unclear. Finally, the long-term safety and toxicity of BTH remain unknown and need further investigation.

Within the limitations of this study, the findings indicate that BTH may be an effective drug delivery system with antibacterial and anti-inflammatory activities for the management of periodontitis. Its thermosensitive formulation is well-suited for application in the gingival sulcus. Thus, it has promising potential as an adjunct to periodontal therapy; however, further research is needed to validate these findings.

## Data Availability

The original contributions presented in the study are included in the article, further inquiries can be directed to the corresponding author.

## Supplementary Materials

The following supporting information can be downloaded at: https://www.mdpi.com/article/10.3390/jfb16080292/s1, Figure S1: Rheological analysis of the novel bakuchiol-loaded thermosensitive hydrogel (BTH) and the vehicle gel; Table S1: Composition of the bakuchiol-loaded thermosensitive hydrogel (BTH) and the vehicle gel; Table S2: Sequences of the primers used for quantitative real-time polymerase chain reaction; Table S3: Gelation temperature and pH of the bakuchiol-loaded thermosensitive hydrogel (BTH) and the vehicle gel.

## Abbreviations

The following abbreviations are used in this manuscript:

BTH	bakuchiol-loaded thermosensitive hydrogel
NSAIDs	nonsteroidal anti-inflammatory drugs
PBS	phosphate-buffered saline
PF-127	Pluronic^®^ F-127
PX188	poloxamer 188
CMC	carboxymethylcellulose sodium salt
SEM	scanning electron microscopy
FTIR	Fourier transform infrared spectroscopy
ATCC	American Type Culture Collection
HGF-1	Human gingival fibroblast
LPS	lipopolysaccharide
PCR	polymerase chain reaction
TNF-α	tumor necrosis factor alpha
IL-1β	interleukin-1 beta
IL-6	interleukin 6
IL-10	interleukin 10
Micro-CT	micro-computed tomography
H&E	hematoxylin and eosin
LCST	lower critical solution temperature
NF-κB	nuclear factor kappa–light-chain-enhancer of activated B cells
