# Supplementary material for: Injectable Thermosensitive Hydrogel Containing Bakuchiol Reduces Periodontal Inflammation and Alveolar Bone Loss in a Rat Model
Source: J Funct Biomater. 2025 Aug 13;16(8):292. doi: 10.3390/jfb16080292 (PMC12387751; doi:10.3390/jfb16080292)
Supplement: Supplementary file 1 [file jfb-16-00292-s001.zip › jfb-3768651-supplementary.pdf]

SUPPLEMENTARY MATERIALS

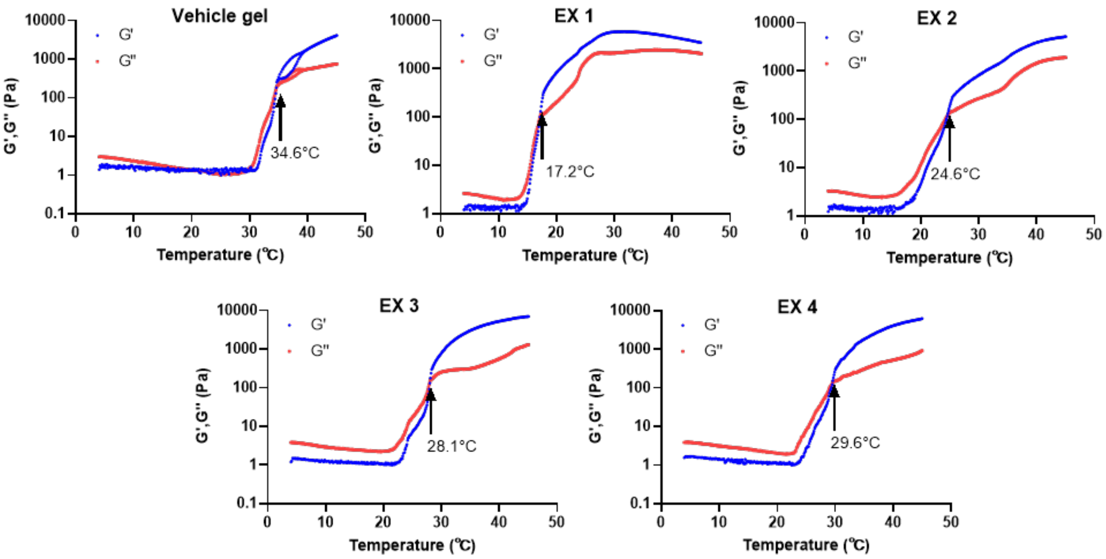

Figure S1. Rheological analysis of the novel bakuchiol-loaded thermosensitive hydrogel (BTH) and the vehicle gel. Temperature-dependent changes in the elastic modulus (G') and viscous modulus (G") were measured using a rheometer to analyze BTH.

Table S1
Composition of the bakuchiol-loaded thermosensitive hydrogel (BTH) and the vehicle gel (unit: g, mL)

	**PF127 (g)**	**P188 (g)**	**CMC (g)**	**Bakuchiol solution (mL)**	**PBS (mL)**
**EX 1**	**3**	**0**	**0.2**	**2**	**18**
**EX 2**	**3**	**0.5**	**0.2**	**2**	**18**
**EX 3**	**3**	**1**	**0.2**	**2**	**18**
**EX 4**	**3**	**1.5**	**0.2**	**2**	**18**
**Vehicle gel**	**3**	**1**	**0.2**	**0**	**20**

PF127: pluronic F-127, P188: poloxamer 188, CMC: carboxymethylcellulose
Bakuchiol solution: 2 mL of bakuchiol dissolved in 10 mL of ethanol and 10 mL of polyethylene glycol solution
PBS: phosphate-buffered saline

Table S2

Sequences of the primers used for quantitative real-time polymerase chain reaction

Primers	Forward	Reverse
M-GAPDH	CTGCACCACCAACTGCTTAG	GTCTTCTGGGTGGCAGTGAT
M-TNF-a	CCACGCTCTTCTGTCTACTG	CTGATGAGAGGGAGGCCATT
M-IL-1b	AAAAAGCCTCGTGCTGTCG	TTTGTCGTTGCTTGGTTCTCC
M-IL-6	AAAGCCAGAGTCCTTCAGAGA	TTGGTCCTTAGCCACTCCTT
M-IL-10	GTGGAGCAGGTGAAGAGTGAT	AGTCCAGCAGACTCAATACACA
R-GAPDH	GGCCTTCCGTGTTCCTA	AAGGTGGAAGAATGGGAGTTG
R-IL-1β	TGTGATGAAAGACGGCACAC	CTTCTTCTTTGGGTATTGTTTGG

M: mouse, R: rat

Table S3

Gelation temperature and pH of the bakuchiol-loaded thermosensitive hydrogel (BTH) and the vehicle gel

Experimental Group	Gelation temperature (°C)	pH
**EX1**	**20**	**7.13±0.06**
**EX2**	**29**	**7.12±0.05**
**EX3**	**33**	**7.09±0.05**
**EX4**	**40**	**7.16±0.02**
**Vehicle gel**	**—**	**7.02±0.05**

— means that no gelation occurred.
